# Responses to the AUDIT questionnaire in the population-based Tromsø surveys as predictor of a diagnosis of alcohol use disorder in Norwegian central health registries—an NCDNOR study

**DOI:** 10.1093/eurpub/ckaf131

**Published:** 2025-10-10

**Authors:** Jørgen G Bramness, Vidar Hjellvik, Wenche Nystad, Anne Høye, Torgeir Gilje Lid

**Affiliations:** Institute of Clinical Medicine, UiT—The Arctic University of Norway, Tromso, Norway; Section for Clinical Addiction Research, Oslo University Hospital, Oslo, Norway; Research Center for Substance Use Disorders and Mental Illness, Innlandet Hospital Trust, Hamar, Norway; Department of Alcohol, Tobacco and Drugs, Norwegian Institute of Public Health, Oslo, Norway; Department of Chronic Diseases, Norwegian Institute of Public Health, Oslo, Norway; Department of Chronic Diseases, Norwegian Institute of Public Health, Oslo, Norway; Department of Clinical Medicine, UiT—The Arctic University of Norway, Tromso, Norway; Center for Clinical Documentation and Evaluation (SKDE), Tromso, Norway; Division of Mental Health and Substance Abuse, University Hospital of North Norway, Tromso, Norway; Centre for Alcohol and Drug Research, Stavanger University Hospital, Stavanger, Norway; Faculty of Health Sciences, University of Stavanger, Stavanger, Norway

## Abstract

Alcohol use and alcohol use disorder (AUD) are major contributors to global morbidity and mortality. The Alcohol Use Disorders Identification Test (AUDIT) is often used for screening of alcohol use and potential alcohol problems, but less is known whether AUDIT can predict a diagnosis of AUD. Responses to the AUDIT questionnaire (*N* = 29 278) from two waves of a population health survey (The Tromsø study) were used to predict a diagnosis of AUD in national health registries over the following three years. Covariates included age, sex, educational level, family income, and mental health score. Overall, 13%–15% scored above the lowest level on AUDIT, with slightly higher figures in males and younger adults, among those with higher education, or with higher mental distress. Few were represented in national health registries (2.1% and 2.7% of these cases in the primary and specialist healthcare, respectively), but with higher figures among those with the highest AUDIT scores. Being female, of older age, having a lower income, and reporting more mental health symptoms increased the probability of receiving an AUD diagnosis. Younger age, male gender, higher education, and higher mental health score predicted higher AUDIT scores, but few, even with high AUDIT scores, were represented in national health registries with an AUD diagnosis. Furthermore, with a high AUDIT score, factors such as older age, lower income, and lower education increased the likelihood of receiving an AUD diagnosis. This suggests that relying on national health registries to monitor alcohol morbidity may be challenging.

## Introduction

The World Health Organization (WHO) has integrated mental health and substance use disorders in its strategy to reduce premature mortality from noncommunicable diseases (NCDs). Severe addiction disorders, especially alcohol use disorder (AUD), constitute a large and possibly growing part of NCDs and represent a heavy disease burden in all regions of the world, particularly in Europe [1–3]. According to the WHO, harmful use of alcohol results in more than 200 diseases and conditions, being responsible for 3 million deaths annually worldwide, representing 5.3% of all deaths [4, 5]. Furthermore, 5.1% of the global burden of disease, as measured in loss of disability-adjusted life years, is attributable to alcohol. These statistics underscore the significant health, social, and economic consequences of alcohol consumption, particularly as it causes death and disability also early in life. It is thus of significance for authorities to keep track of alcohol use and alcohol problems in the society.

Alcohol use and negative consequences following alcohol use vary with sex [6], mental and physical health, and socioeconomic status (SES). The relationship between alcohol consumed and related problems is more pronounced among those with lower SES, even if higher SES is often associated with higher alcohol consumption levels [7]. In other words, we observe a negative social gradient in harms from alcohol despite a positive social gradient in alcohol exposure, a phenomenon called the “alcohol harm paradox”.

Alcohol use is often not addressed by health care workers as a risk factor for disease [8]. AUD is often unrecognized, undiagnosed, and untreated [9]. The time from debut to treatment may be as much as two decades [10]. Earlier treatment could have benefited the patients. There are several reasons for not seeking help for AUD, both personal and system related [11], including unawareness of treatments offered, economic barriers, low belief in the help being offered, the stigma related to AUD, and a belief that the problems will resolve without intervention or that one will be able to handle it alone [12].

The Alcohol Use Disorder Identification Test (AUDIT) was intended for primary health care as part of a screening and brief intervention procedure [13]. However, AUDIT has not been successfully implemented [14]. There is also the question of how well AUDIT and other screening instruments measure true alcohol consumption, with a discrepancy between real and reported use of 40%–60% [15]. Underestimation is more marked for young males, in middle-aged females and in those who engaged less often in heavy drinking [16].

Utilizing the AUDIT questionnaire in population-based studies may thus be problematic. Lastly, there can be a selection bias as high alcohol use and AUD may drive non-response in such studies. Previous studies have shown that alcohol use and mental distress are moderately associated with non-response [17], though probably not a major cause, as controlling for other variables weakened the associations [18].

We have recently shown that the national health registries may be used to follow the population health status concerning depressive disorders, albeit with low representation [19]. This finding solved some of the problems related to the costs of administering large population surveys to investigate depression in the population. Given the challenges in detecting and monitoring alcohol use and alcohol use disorder, we wanted to pursue a similar approach for alcohol. We use a unique combination of population-based health surveys and national health registries to see if these registries also could be used to monitor the alcohol-related morbidity in the population. Furthermore, we address the following research questions:

- What does the general population report on their alcohol use and are there differences in self-reported alcohol use between two surveys conducted in different time periods or between sex, age, SES, or with level of psychological distress?
- Does the relationship between self-reported alcohol use and a diagnosis of alcohol use disorder in national health registries vary with time or by sex, age, SES, or level of psychological distress?

## Methods

### Exposure data

Data were obtained from two waves of the Tromsø study: Tromsø6 (2007–08) and Tromsø7 (2015–16). These studies were comprehensive general population health surveys conducted in urban and rural areas of northern Norway, targeting individuals aged 40 years or older. Data on sex distribution, number of invitees, and response rate are presented in Supplementary Table S1. The background variables on SES from Statistics Norway were linked to the health survey data using the encrypted Norwegian 11-digit unique person-identifier. These variables included level of education (compulsory, intermediate, higher) and level of income (total income from work, capital, and welfare benefits rounded to nearest 10 000 NOK). Income was grouped into four categories by quantiles within each 10-year age group and sex (*q*_0-10_, *q*_10-40_, *q*_40-70_, and *q*_70-100_), where those with an income equal to the highest limit were placed in the group below. The most deviating results concerning alcohol use and representation in national health registries were seen in the lowest income range, motivating the unequal group sizes.

The main exposure variable was the informants’ responses on the AUDIT questionnaire from both waves quantifying their alcohol consumption and alcohol-related problems during the last year. Responses to the 10 AUDIT questions were added together in four categories: low risk alcohol consumption (seven or less for males or five or less for females); moderate risk alcohol consumption (8–15 for males or 6–15 for females); high-risk alcohol consumption with a score of 16–19 for both sexes; and scores of 20 or more were categorized as ultra-high risk alcohol consumption. Only individuals who responded to all AUDIT questions were included. The only exception was for persons who reported no alcohol use as response to the first question. These were included as scoring zero, regardless of their responses to the other nine AUDIT questions.

We used Hopkins Symptom Checklist (HSCL) 10-items version to measure psychological distress [20]. Symptoms related to anxiety and depression over the past week were scored on a scale from 1 (not at all) to 4 (extremely). The mean score was calculated producing a range of scores from 1 to 4, where higher score corresponds to more psychological distress. An average score ≥1.85 has commonly been considered a cut-off to identify psychological distress [21]. For this study, the five items covering depression were included, and only those responding to all five were included. The average score was dichotomized as low (≤1.8) and high (≥2).

### Outcome data

The data from the two waves of the Tromsø study were linked with data from Norwegian Control and Payment of Health Reimbursements Database (CPHR) 2006–2020 and the Norwegian Patient Registry (NPR) 2008–2020. Codes for identification of outcome and their interpretation are given in Supplementary Table S2. We searched for representation in the registries within the time window of 0–1095 days following the date of each wave of the health survey.

The CPHR includes a primary health care database that registers all reimbursement claims from primary care physicians in Norway. This database has full national coverage of nearly all patient contacts in primary health care, as about 98% of the population was registered with a primary care physician included in CPHR in the study period, and very few private alternatives existed. Representation in CPHR was defined as having a diagnosis of AUD in either ICPC-2 (P15 or P16) or ICD-10 (F10) at least once.

The NPR covers all treatment of patients in specialist health care in Norway, with full national coverage. Representation in NPR was defined as having an ICD-10 F10 diagnosis at least once.

### Data analyses

First, the number of survey participants in each of the four AUDIT categories was computed for each stratum of sex, age (40–49, 50–59, 60–79), income, and HSCL score (low, high). Then, for each combination of the above listed exposure and AUDIT categories, the proportion that were represented in NPR or CHPR during 0–1095 days following the date of each health survey was calculated.

This a sub-study in the project ‘A life-course approach to prevent noncommunicable diseases in an aging population—NCDNOR’, approved by the Regional Committee for Medical and Health Research Ethics of South-Eastern Norway 2019/1203. Informed consent was obtained from all those participating in the general population surveys. The research was conducted in accordance with the Declaration of Helsinki.

## Results

Among the 29 278 responses to the two waves of the Troms study, most people scored low on the AUDIT questionnaire, with less than 1% having a high or ultra-high risk alcohol consumption (Table 1). More people reported moderate- to high-risk alcohol consumption in the second wave of the study. There was an increase from 14.8% to 18.2% for men and 10.0% to 13.0% for women. This represent an approximately 25% increase, but apart from this, there were no major differences between the two waves regarding AUDIT score. Males reported more high-risk alcohol consumption than females. The oldest adults reported less high-risk alcohol consumption compared to the young adults. The higher income groups reported more moderate risk alcohol consumption, while the lower income groups more often reported low risk alcohol consumption. The same tendency was seen for educational levels; the higher education group reported more moderate risk alcohol consumption. A score above the cut-off for HSCL was related to high and ultra-high risk alcohol consumption.

**Table 1.** The score on AUDIT and number of respondents in Tromsø study wave 6 and 7, stratified on sex, age, income, education, and HSCL score^a^

	Tromsø6					Tromsø7				
		AUDIT category					AUDIT category			
	Total	Low	Moderate	High	Very high	Total	Low	Moderate	High	Very high
	*N* (%)	*N* (%)	*N* (%)	*N* (%)	*N* (%)	*N* (%)	*N* (%)	*N* (%)	*N* (%)	*N* (%)
	10 499	9140 (87.1)	1274 (12.1)	54 (0.5)	31(0.3)	18 779	15 871 (84.5)	2727 (14.5)	119 (0.6)	62 (0.3)
Sex										
Male	5005 (47.7)	4193 (45.9)	742 (58.2)	47 (87.0)	23 (74.2)	8989 (47.9)	7357 (46.4)	1483 (54.4)	98 (82.4)	51 (82.3)
Female	5494 (52.3)	4947 (54.1)	532 (41.8)	7 (13.0)	8 (25.8)	9790 (52.1)	8514 (53.6)	1244 (45.6)	21 (17.6)	11 (17.7)
Age										
40–49	3106 (29.6)	2476 (27.1)	591 (46.4)	26 (48.1)	13 (41.9)	5712 (30.4)	4561 (28.7)	1093 (40.1)	35 (29.4)	23 (37.1)
50–59	2206 (21.0)	1855 (20.3)	326 (25.6)	19 (35.2)	6 (19.4)	5762 (30.7)	4756 (30.0)	936 (34.3)	51 (42.9)	19 (30.6)
60–79	5187 (49.4)	4809 (52.6)	357 (28.0)	9 (16.7)	12 (38.7)	7305 (38.9)	6554 (41.3)	698 (25.6)	33 (27.7)	20 (32.3)
Income										
*q*_0-10_	1154 (11.0)	1014 (11.1)	114 (8.9)	15 (27.8)	11 (35.5)	2008 (10.7)	1702 (10.7)	263 (9.6)	27 (22.7)	16 (25.8)
*q*_10-40_	3208 (30.6)	2798 (30.6)	374 (29.4)	23 (42.6)	13 (41.9)	5719 (30.5)	4829 (30.4)	827 (30.3)	38 (31.9)	25 (40.3)
*q*_40-70_	3118 (29.7)	2721 (29.8)	385 (30.2)	16 (29.6)	7 (22.6)	5558 (29.6)	4754 (30.0)	767 (28.1)	23 (19.3)	14 (22.6)
*q*_70-100_	3013 (28.7)	2602 (28.5)	400 (31.4)			5494 (29.3)	4586 (28.9)	870 (31.9)	31 (26.1)	7 (11.3)
Education										
Low	2391 (22.8)	2138 (23.4)	234 (18.4)	13 (24.1)	6 (19.4)	2861 (15.2)	2471 (15.6)	363 (13.3)	17 (14.3)	10 (16.1)
Mid	4685 (44.6)	4089 (44.7)	558 (43.8)	23 (42.6)	15 (48.4)	7511 (40.0)	6383 (40.2)	1059 (38.8)	48 (40.3)	21 (33.9)
High	3412 (32.5)	2903 (31.8)	481 (37.8)	18 (33.3)	10 (32.3)	8355 (44.5)	6970 (43.9)	1301 (47.7)	53 (44.5)	31 (50.0)
HSCL										
Low (1–1.84)	8893 (84.7)	7767 (85.0)	1078 (84.6)	34 (63.0)	14 (45.2)	16 264 (86.6)	13 957 (87.9)	2202 (80.7)	74 (62.2)	31 (50.0)
High (≥1.85)	924 (8.8)	742 (8.1)	147 (11.5)	18 (33.3)	17 (54.8)	2074 (11.0)	1510 (9.5)	490 (18.0)	43 (36.1)	31 (50.0)
In register										
No	10 456 (99.6)	9126 (99.8)	1259 (98.8)	47 (87.0)	24 (77.4)	18 674 (99.4)	15 845 (99.8)	2686 (98.5)	104 (87.4)	39 (62.9)
Yes	43 (0.4)	14 (0.2)	15 (1.2)	7 (13.0)	7 (22.6)	105 (0.6)	26 (0.2)	41 (1.5)	15 (12.6)	23 (37.1)

a The table also includes the representation in the health registries with an AUD diagnosis. Due to low figures, some cells are collapsed. Income is the total income (from work, capital, and benefits) in 2006 for Tromsø6 and 2014 for Tromsø7 split by the 10%, 40%, and 70% quantiles within each 10-year age group and sex (incomes equal to the 10%, 40%, and 70% quantiles are placed in the *q*_0-10_, *q*_10-40_, and *q*_40-70_ group, respectively).

Of all the respondents to the surveys, only 148 people (0.5%) were found in either primary or specialist health registries with a diagnosis of AUD during the follow-up (Table 2). There was a dose–response relationship such that the representation in the national health registries increased with increasing alcohol use risk. Specifically, 0.2% of the responders with low risk were found with an AUD diagnosis in national health registries, 1.4% of those with moderate risk, 12.7% of those with high-risk, and 32.2% of those with ultra-high risk.

**Table 2.** Number and proportion of those who report moderate drinking or more on AUDIT in the population surveys who are found in different central health registries 0–1095 days after symptom scores^a^

	Tromsø6						Tromsø7					
		AUDIT score	In registries 0–1095 days after survey *N* (% of above moderate or more)			AUDIT score		AUDIT score	In registries 0–1095 days after survey *N* (% of above moderate or more)			AUDIT score
	*N* total	*N* (%) (moderate or more)	KUHR	NPR	ANY	*N* (%) (high or more)	*N* total	*N* (%) (moderate or more)	KUHR	NPR	ANY	*N* (%) (high or more)
All	10 499	1359 (11.5)	20 (1.5)	20 (1.5)	29 (2.1)	85 (6.3)	18 779	2908 (13.4)	51 (1.8)	59 (2.0)	79 (2.7)	181 (6.2)
Sex												
Male	5005	812 (14.0)	13 (1.6)	15 (1.8)	20 (2.5)	70 (8.6)	8989	1632 (15.4)	32 (2.0)	38 (2.3)	49 (3.0)	149 (9.1)
Female	5494	547 (9.1)	7 (1.3)	5 (0.9)	9 (1.6)	15 (2.7)	9790	1276 (11.5)	19 (1.5)	21 (1.6)	30 (2.4)	32 (2.5)
Age												
40–49	3106	630 (16.9)	10 (1.6)	7 (1.1)	12 (1.9)	39 (6.2)	5712	1151 (16.8)	17 (1.5)	16 (1.4)	23 (2.0)	58 (5.0)
50–59	2206	351 (13.7)	5 (1.4)	7 (2.0)	9 (2.6)	25 (7.1)	5762	1006 (14.9)	14 (1.4)	17 (1.7)	24 (2.4)	70 (7.0)
60–79	5187	378 (6.8)	5 (1.3)	6 (1.6)	8 (2.1)	21 (5.6)	7305	751 (9.3)	20 (2.7)	26 (3.5)	32 (4.3)	53 (7.1)
Income^b^												
*q*_0-10_	1154	140 (10.8)	7 (5.0)	7 (5.0)	12 (8.6)	26 (18.6)	2008	306 (13.2)	21 (6.9)	25 (8.2)	34 (11.1)	43 (14.1)
*q*_10-40_	3208	410 (11.3)	6 (1.5)	6 (1.5)	7 (1.7)	36 (8.8)	5719	890 (13.2)	18 (2.0)	20 (2.2)	27 (3.0)	63 (7.1)
*q*_40-70_	3118	397 (11.3)	7 (0.9)	7 (0.9)	10 (1.2)	12 (3.0)	5558	804 (12.6)	6 (0.7)	6 (0.7)	9 (1.1)	37 (4.6)
*q*_70-100_	3013	411 (12.0)				11 (2.7)	5494	908 (14.2)	6 (0.7)	8 (0.9)	9 (1.0)	38 (4.2)
Education^b^												
Low	2391	253 (9.6)	15 (1.8)	15 (1.8)	6 (2.4)	19 (7.5)	2861	390 (12.0)	10 (2.6)	10 (2.6)	16 (4.1)	27 (6.9)
Mid	4685	596 (11.3)			17 (2.9)	38 (6.4)	7511	1128 (13.1)	21 (1.9)	28 (2.5)	36 (3.2)	69 (6.1)
High	3412	509 (13.0)	5 (1.0)	5 (1.0)	6 (1.2)	28 (5.5)	8355	1385 (14.2)	20 (1.4)	21 (1.5)	27 (1.9)	84 (6.1)
HSCL^b^												
Low (1.0–1.8)	8893	1126 (11.2)	12 (1.1)	12 (1.1)	17 (1.5)	48 (4.3)	16264	2307 (12.4)	24 (1.0)	26 (1.1)	37 (1.6)	105 (4.6)
High (2.0–4.0)	924	182 (16.5)	8 (4.4)	7 (3.8)	11 (6.0)	35 (19.2)	2074	564 (17.3)	26 (4.6)	31 (5.5)	40 (7.1)	74 (13.1)

a HSCL: Hopkins Symptom Checklist; HADS: Hospital Anxiety and Depression Scale; CPHR: Norwegian Control and Payment of Health Reimbursements Database; NorPD: Norwegian Prescription Database; NPR: Norwegian Patient Registry.

b *N* with missing education: 11 (TU6), 56 (TU7); *N* with missing income: 6 (TU6), 0 (TU7); *N* with missing HSCL: 682 (TU6), 441 (TU7).

**Figure 1.**
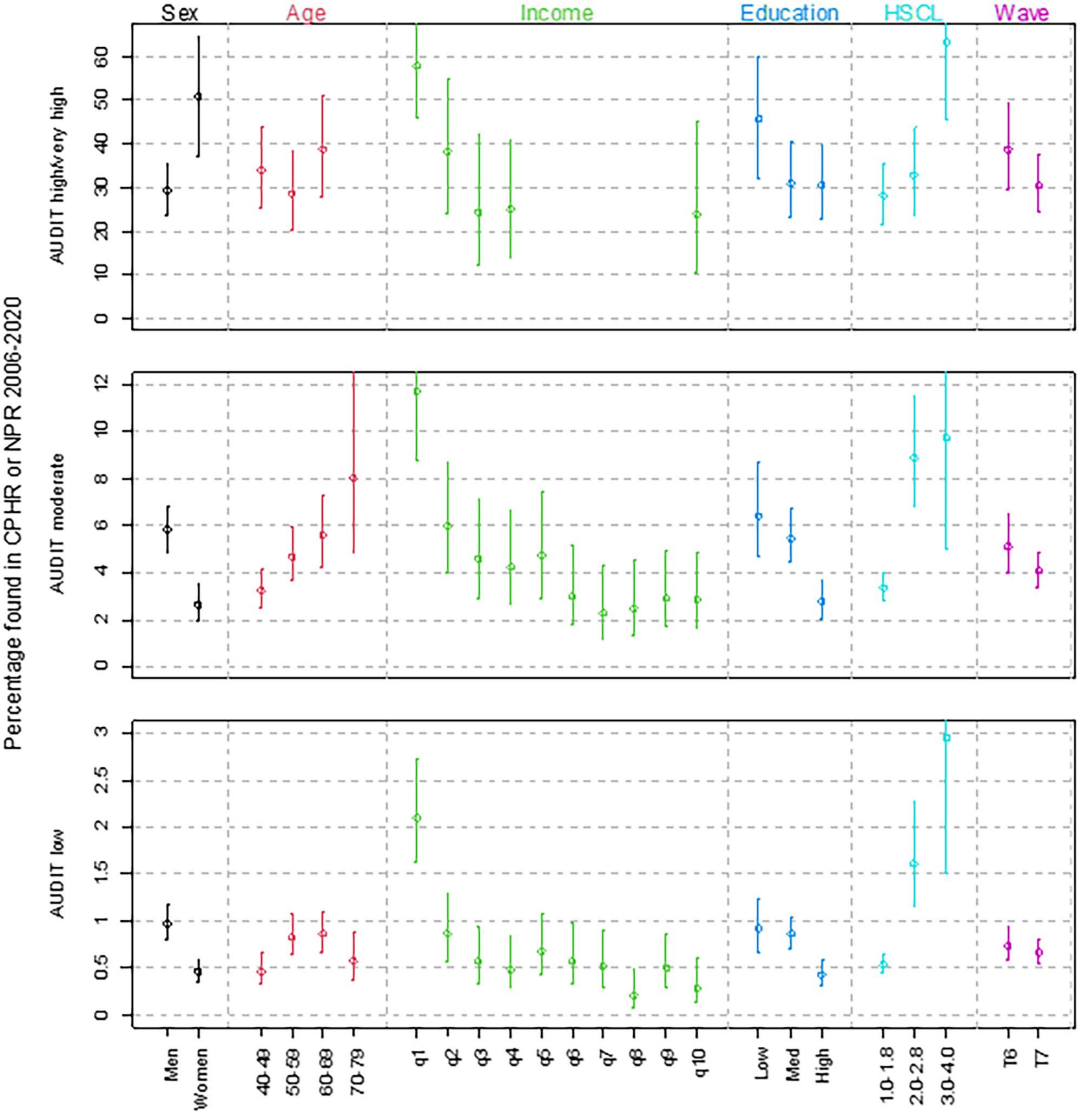
Proportion (95% CI) of those with low, moderate, and high/very high AUDIT score in Tromsø6 and 7, who were registered in CPHR 2006–2020 with an ICPC-2 diagnosis of P15 or P16 or an ICD-10 F10 diagnosis or who were registered in the NPR 2008–2020 with an ICD-10 F10 diagnosis stratified on sex, age at date of survey, income,* and education in 2006 and 2014 for Tromsø6 and 7, respectively, education, HSCL score, and wave (Tromsø6 or 7). Strata with <5 persons found in KUHR/NPR are not shown. *Total income in deciles within each sex- and 10-year age group.

Of those who reported above low risk alcohol consumption, slightly more were represented in national health registries in Tromsø7 compared to Tromsø6. There were no major differences in how many were diagnosed in primary or specialist health care. However, these registries did not completely overlap, as being registered in either of the two resulted in a slightly higher figure than being registered in just one of them. Males reporting moderate risk alcohol use or above were registered slightly more often in the health registries than females with the same risk profile (Fig. 1). There was also a minor age effect, where elderly with this score were more frequently represented in the health registries. Low SES, as measured by a low income, and even more so by low education, resulted in far more diagnoses of AUD in national health registries. Lastly, a score above cut-off on HSCL resulted in far more diagnoses of AUD in national health registries.

## Discussion

In these general population health surveys on self-reported alcohol use using the AUDIT, we found that most people reported low risk alcohol consumption with only a moderate increase in self-reported alcohol use between the two waves of the study. High-risk self-reported alcohol consumption was related to male sex, lower age, and higher depressive score. By linking these survey data to national health registries, we found that very few people with a moderate or even high-risk alcohol consumption had an AUD diagnosis in these. There was, however, a dose–response relationship between self-reported alcohol consumption and the risk of receiving an AUD diagnosis in national health registries from either primary or specialist health care. The proportion of individuals later diagnosed with AUD increased in the most recent wave of the study. Despite a decrease in self-reported alcohol use with age, older people with high AUDIT scores more often had an AUD diagnosis in health registries. Males with high AUDIT scores were only slightly more represented than females. We found few differences in alcohol use across educational groups, but those with lower educational levels more often had an AUD diagnosis in national heath registries. A high score on depressive symptoms increased the risk of high-risk alcohol consumption, and for being registered with a diagnosis of AUD in the national health registries.

Our study population was responders to the surveys. We cannot evaluate non-response among AUD patients. However, at least some responders received a subsequent AUD diagnosis. This corresponds with earlier studies showing that alcohol use and mental distress are only moderately associated with non-participation [18]. Additionally, we know that people generally underreport their alcohol use in such self-report studies [16]. Nevertheless, the present study demonstrates that the ‘relationship’ between known risk factors reflected what we know from other studies. For example, the current study reproduced findings from other studies that male sex [22], younger age [23], and higher depressive symptoms [24] are related to higher alcohol use.

Despite the dose–response relationship between AUDIT scores and representation in national health registries with a diagnosis of AUD, very few, even among those with the highest scores, had an AUD diagnosis in these registries. This may reflect the underdiagnosis and undertreatment of AUD, with as few as 5%–7% of those identified as having an AUD in diagnose-based population surveys reappearing in national heath registries [9]. Such underdiagnosing is similar to what is found also for other disorders due to various causes. For example, myalgic encephalomyelitis (ME) is often underdiagnosed due to difficult and uncertain diagnostic criteria [25, 26], schizophrenia may be underdiagnosed due to the severity of the disorder and its life-long and complex symptom trajectories [27], and dementia is frequently underdiagnosed due to the lack of a cure and challenges in detection and communication [28]. Undertreatment may be driven by factors related to the treatment system and may also be due to the patient’s behaviour. Doctors often hesitate to ask patients about their alcohol use as it is seen as a personal and sensitive issue, thus missing the chance for intervention [29]. Additionally, the doctors may lack knowledge about interventions or lack trust in the access to specialized treatment, and thus refrain from the topic [25]. Patients may have a low perception of need, be unaware of the problem or of treatments offered and there may be financial barriers, low trust in the help offered, issues related to the stigma of AUD, or a belief that the problem will resolve without intervention [12]. An additional factor may be that a high score on the screening instrument AUDIT does not equal a diagnosis of AUD. AUDIT has varying sensitivity and specificity in different populations and settings [30].

We observed an increase in those reporting moderate- to high-risk alcohol consumption. This is known from earlier research [31]. The increase in the share of responders being given a later AUD diagnosis can be explained by this increase, thus revealing no major change in rate of diagnosis between the waves.

Older individuals with higher AUDIT scores were more frequently diagnosed with an AUD in national health registries. This may reflect delays caused by doctors and patients, influenced by many of the same factors that explain the undertreatment mentioned previously. Also, it takes time to develop an alcohol problem, maybe contributing to age increasing the chance of receiving an AUD diagnosis [32]. Lastly, older people have more health issues and seek medical care more often, which should result in a higher detection rate of AUD.

Men with high AUDIT scores were more often diagnosed with an AUD. AUD is often regarded as a ‘male disorder’. Even though women seek healthcare more frequently than men, a Norwegian population-based survey found that females less often experienced alcohol being addressed in contact with health care [33]. Many of the aforementioned factors, such as a low perception of need, can contribute to women underestimating their need for treatment. Additionally, feelings of guilt and shame may further prevent women from seeking help more than men [34].

Despite few differences in alcohol use across educational levels, more respondents with lower education had a diagnosis of AUD in the national health registries. This ‘alcohol harm paradox’ states that despite an overall straightforward dose–response relationship between alcohol consumption and alcohol-related problems, the problematic use is more pronounced among those with lower SES [7]. In other words, one may observe a negative social gradient in harms from alcohol despite a positive social gradient in alcohol exposure. Furthermore, the physicians’ attitudes towards patients with different SES may also play a part. A qualitative study from UK found that primary care physicians’ own drinking practices and social status may act as a benchmark, making it easier to recognize risk drinking in patients not resembling themselves [35]. In addition, people from higher SES may seek more private health care, a sector that do not report to NPR.

People who together with high AUDIT scores also had high scores on HCSL-10 were more often found in the national health registries with an AUD diagnosis. Generally, people with depression more often have alcohol problems [36]. However, this higher representation of an AUD diagnosis in national health registries may also reflect that increased mental distress may heighten people’s need for help and lower their barriers to seeking it. Additionally, health care workers’ recognition of the person’s need for help can contribute to clearer communication and the implementation of appropriate measures.

### Limitations

In the current study, we lack a gold standard of who has AUD. This could only be determined through diagnosis-based population surveys. Very few such surveys have ever been conducted in Norway, and we are aware of only one that related such a study to the national health registries [9]. Such studies are extremely costly and difficult to perform, and the current study was designed to explore an alternative approach. We have previously used population-based surveys with self-rated depression measures as a gold standard and argued that central health registries can be used to track trends in depression in different groups and over time [19]. However, for depression, the prevalence in the national health registries was much higher than that for AUD in the current study. Furthermore, our data were limited, with scarce information. Also, our study catches only diagnoses given within the public sector. Some people will seek help for their alcohol use problems in private clinics, which are not captured in the current study. However, private caregivers are limited in Norway and are likely to introduce only a small bias. Taken together, the study captures the entire Norwegian adult population within a study area, likely making it representative of this cohort.

## Conclusion

This study coupled data from two general population health surveys and two national health registries for primary and specialized health care to see if high scores on AUDIT was related to being registered in these registries with AUD. To the best of our knowledge, this combination of data is unique. We found that younger age, male gender, higher education, and higher mental health score predicted higher AUDIT scores. There was also a marginal increase from the first to the last wave in those reporting higher scores. The main conclusion in our study is that very few of those who reported an AUDIT score of moderate risk or more were found in the central health registries, implying that it is difficult to use information from central health registries to assess alcohol use problems at the population level. This finding contrasts from a conclusion we drew from a similar study on self-reported depressive symptoms and central health registries [19]. Rather large discrepancies between different investigated strata adds to these reservations.

## Supplementary Material

ckaf131_Supplementary_Data

## Data Availability

The datasets used and/or analysed during this study are available from the corresponding author, only after application has been made to and permission given by the ethics committee. Key points Despite a positive dose–response relationship between AUDIT scores and the likelihood of an AUD diagnosis in health registries, very few individuals, even with high AUDIT scores, received an AUD diagnosis. Younger age, male gender, higher education, and higher mental health scores predicted higher AUDIT scores. With a high AUDIT score, factors such as older age, lower income, and lower education were more strongly associated with receiving an AUD diagnosis. The study highlights the challenges of using central health registries alone for monitoring alcohol morbidity in the population. Public health policies and practices should focus on improving early detection and treatment of AUD, particularly for those with high levels of mental distress or low socioeconomic status.
