# Supplementary material for: Responses to the AUDIT questionnaire in the population-based Tromsø surveys as predictor of a diagnosis of alcohol use disorder in Norwegian central health registries—an NCDNOR study
Source: Eur J Public Health. 2025 Oct 10;35(6):1219–25. doi: 10.1093/eurpub/ckaf131 (PMC12707466; doi:10.1093/eurpub/ckaf131)
Supplement: ckaf131_Supplementary_Data [file ckaf131_supplementary_data.docx]

**Supplementary table S1.** Overview over the included population surveys utilized in the current study.

|  | Years performed | Age range | Screening instrument used | Number 40-69 years of age | Number with complete data |
| --- | --- | --- | --- | --- | --- |
| Tromsø6 | 2007-08 | 40-79 years of age | AUDIT | 11899 | 10499 (88.2%) |
| Tromsø7 | 2015-16 | 40-79 years of age | AUDIT | 21082 | 18799 (89.2%) |

**Supplementary table S2.** Overview over the included codes from central health registries.

|  | Codes | Interpretation/wording |
| --- | --- | --- |
| *Norwegian Registry for Primary Health Care (NRPHC)* | | |
| ICPC-2 | P15, P16 | Acute alcohol intoxication and alcohol problems |
| ICD-10 | F10 | Any alcohol use disorder |
| *Diagnosis represented in Norwegian Patient Registry (NPR)* | | |
| ICD-10 | F10 | Any alcohol use disorder |
